# Pollution of the sediments of the coastal zone of the Sambia Peninsula and the Curonian Spit (Southeastern Baltic Sea)

**DOI:** 10.7717/peerj.4770

**Published:** 2018-05-16

**Authors:** Alexander Krek, Viktor Krechik, Aleksandr Danchenkov, Elena Krek

**Affiliations:** 1 Shirshov Institute of Oceanology, Russian Academy of Sciences, Moscow, Russia; 2 Immanuel Kant Baltic Federal University, Kaliningrad, Russia

**Keywords:** Sediments, Pollution indexes, Coastal zone, Baltic Sea, Heavy metals

## Abstract

The detailed environmental survey of the coastal zone of the Kaliningrad Region northern coast was carried out. The pollutants distribution in the silty clay fraction and calculation of ecological indexes allowed the evaluation of distribution of potentially harmful elements (PHEs). The sources of pollution in the most intensively used areas were identified, and transit and accumulation zones were allocated. A large area of anomalous content of PHEs was revealed on the underwater coastal slope of the Curonian Spit National Park, which is situated far from the sources of pollution. The alongshore bed load transport provides the contamination of the underwater slope whereas the beaches are less exposed to pollution.

## Introduction

The Baltic Sea is one of the most polluted water bodies in the world (Kautsky & Kautsky, 2000; HELCOM, 2003; HELCOM, 2010; Garnaga, 2012; HELCOM, 2018). The high anthropogenic load within its catchment area leads to a large number of hazardous substances entering the water area with a river runoff (Cox & Preda, 2005; Gonzalez-Mendoza, Moreno & Zapata-Perez, 2007; Dias et al, 2009; Garnaga, 2012; Yan et al., 2015). Arriving in the marine environment, the substance undergoes physical, chemical, and biological separation (Emelyanov, 1998). That is why the coastal zone is the most susceptible to pollution. Unlike deep sub-basins, the coastal zone experiences high energy and sediment accumulation dynamics as long as a process variety, occurring from the waves and currents interaction, which is formed under the influence of a local atmosphere circulation (Dolotov, 1989; Aybulatov, 1990). This fact complicates the pollution measurement and identification of its sources significantly.

Area studied could be divided into two parts: northern coast of Sambia Peninsula and Russian part of the Curonian Spit. The first one is a densely populated area with several towns and high economic activity (port, industrial enterprises, transportation network, wastewater discharges and others). However, the Curonian Spit is a National Park, a UNESCO World Heritage Site, and there is no significant potential sources of pollution.

Among the most dangerous marine ecosystem pollutants there are oil products (OP) and heavy metals (HM) (Gerlach, 1981; Nemerow, 1991; Leivuori, 2000; Swedish EPA, 2000; Trofimov & Ziling, 2002; DeMora et al, 2004; HELCOM, 2010). The content of the most significant HM (Hg, Cd, Cu, Ni, Pb, Zn) and OP in the clastic sediments were measured in the coastal zone of the Sambia Peninsula northern coast and the Russian part of the Curonian Spit. The purpose of this research was to determine both the pollution level of the coastal zone and the sources of HM and OP in it, to identify the sources of pollution, and to trace its migration routes.

## Materials and Methods

### Sampling

**Figure 1:**
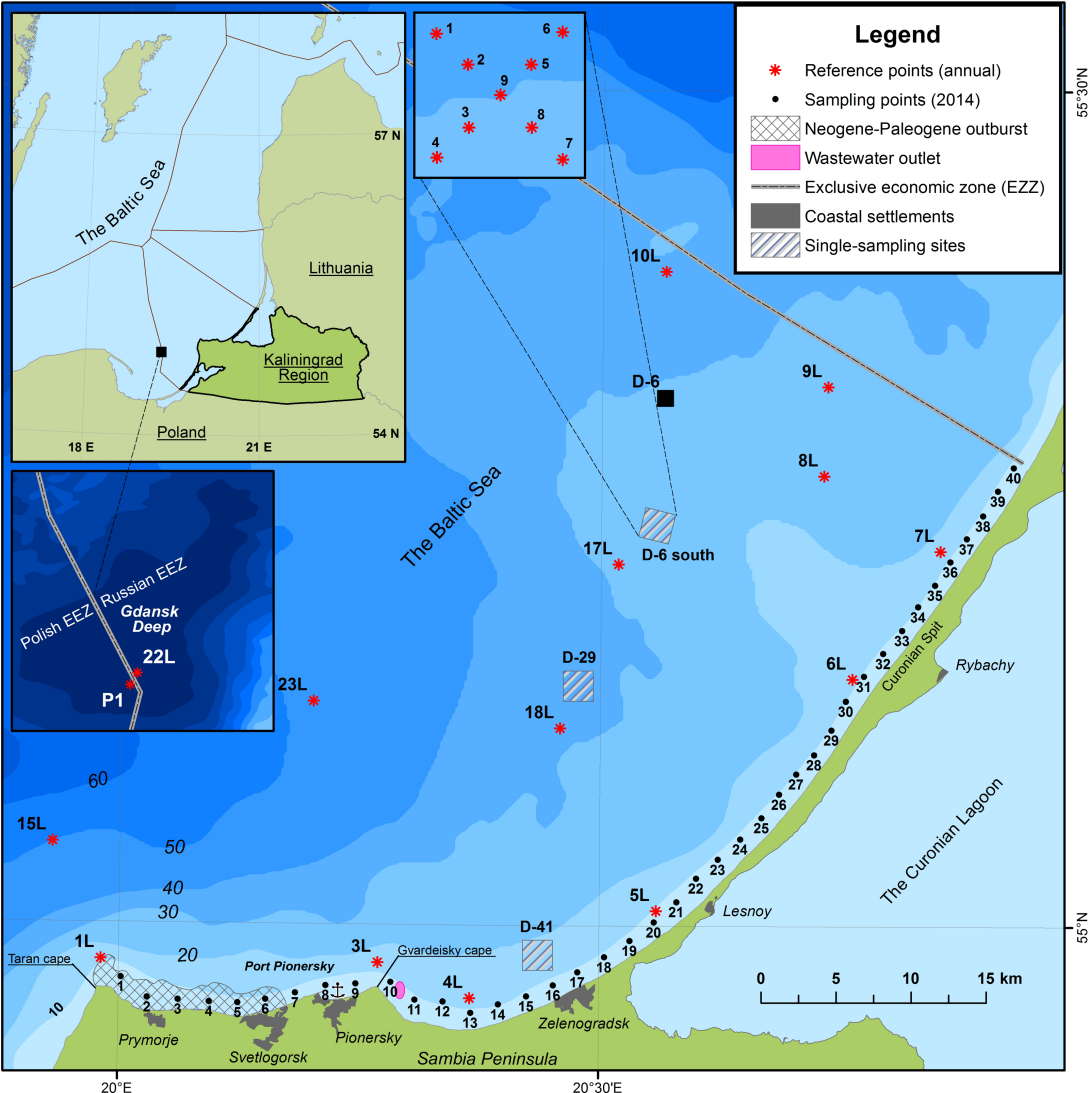
Initial data map.

Surface sediment samples (0–5 cm) from the underwater coastal slope and beaches were taken within a regular profile grid with 2 km interval (Fig. 1). Bottom sediments were taken at points on each profile from 10 m depth by a Van-Veen grab sampler in May 2014. The beach sediments were sampled in June 2014 by a composite technique of samples collected from a beach profile from water’s edge to its back. As a result, an average beach sample consisting of 5–7 point samples, depending on the width of the beach was obtained. To identify possible sources of contamination, soil samples (sandy loam soil remoted from industrial enterprises) of the coastal settlements (Primorye, Otradnoye, Svetlogorsk, Pionersky, Zaostrovye, Lesnoy) were taken. After sampling, the samples were sent to the laboratory for analysis. For the primary estimation of the quantitative content of pollution, the background content of HM (Cd, Cu, Pb, Hg) and OP obtained for clastic sediments (a total of 158 samples obtained during 2011–2015) (see Fig. 1) were used. The authors took the average HM concentration in sediments as a long-standing natural-anthropogenic background. Most countries (including Russia) do not have environmental quality standards for sediments set in legislation. In these cases, it is recommended to use guideline values mentioned in the Working Group report on Marine Sediments in Relation to Pollution (WGMS, 2003) for a general assessment of bottom sediments contamination.

### Laboratory analysis

The grain size analysis of bottom sediments was carried out by the sieve scattering method. When sieving, the Krumbein phi scale was used (Krumbein, 1934) with the following sieves: 4.0; 2.8; 2.0; 1.4; 1.0; 0.71; 0.5; 0.355; 0.25; 0.18; 0.125; 0.09; 0.063; and 0.05 mm.

The content (total) of Pb, Cd, Cu, Ni, Zn was determined by the method of atomic emission spectrometry, Hg by the method of flameless atomic adsorption, OP by infrared spectrometry.

Samples for HM (Cd, Cu, Ni, Pb, Zn) analysis were initially dried naturally in the air in a controlled clean environment for a week. Then, the samples were transferred to an oven and dried at 40 ± 2 °C. The samples were then ground to a powder with a mortar and pestle and kept in a pre-cleaned container for future use. Total digestion of an aliquot of 0.5 g sediment samples was performed in Teflon beakers using 5 ml HNO_3_ + 5 ml HCl + 1 ml HF + 10 ml H_2_O at 210 °C with pressure 175 bar in a first stage and using 30 ml H_3_BO_3_ (4%) in second stage. The reagents were added to the weighted sample, and then placed in a reaction microwave oven. After cooling, the resulting solution was filtered using filters with a pore size of 1–2.5 nm, dosed to 100 ml with bidistilled water. The resulting solution was analyzed. Sample solutions and reagent blanks were analyzed for metals of interest using OPTIMA 2100 DV (ICP-OES method). Instrument detection limits was 0.607 × 10^−3^ for Cd, 1.41 × 10^−3^ for Cu, 0.839 × 10^−3^ for Ni, 2.21 × 10^−3^ for Pb, 1.19 × 10^−3^ for Zn. Background correction and matrix interference were monitored throughout the analyses. According to accredited methods in the Russian Federation, the protocols recorded values of at least 0.05 for Cd, 0.5 for Cu, 0.5 for Ni, 0.5 for Pb, 0.5 for Zn. All values are given in mg/kg.

Hg analysis was performed using RA-915+ Zeeman mercury analyzer (LUMEX Ltd., Solon, OH, USA) with detection limits of 5 × 10^−3^ mg/kg. Samples for OP analysis were initially dried naturally in the air in a controlled clean environment. Organics parts was manually removed. Sediments solute for OP extraction was prepared using 5 g of sediments sample + 10 ml CCl_4_. Extraction from the sample was carried out three times, with subsequent integration of the extract into a flask. The extract is filtered through a 5 µm filter and then analyzed using AN-2. Sensors for concentration measurement has detection limit 40 mg/kg.

### Preliminary analysis

The difference in the grain size composition of bottom sediments within the coastal zone does not allow to estimate the contamination of various parts of the area with sufficient reliability. HM and OP pollutants are associated with silty clay fraction (less than 0.063 mm) (Szefer et al., 1995; Pempkowiak et al., 1998; Pempkowiak, Sikora & Biernacka, 1999; Beldowski & Pempkowiak, 2003; Zaborska, Winogradow & Pempkowiak, 2014) due to its high sorption capacity (McCave, 1984; Nemirovskaya, Ulyanova & Sivkov, 2014). Its content in each sample significantly differed, so the authors suggested that its content in the sample would strongly affect the PHEs content. To compare the obtained results they were normalized to this fraction, by the ratio of the PHEs concentration in the sample to the percentage of particles less than 0.063 mm in diameter. Subsequently, such an approach allowed estimating the contribution of known sources to the PHEs distribution along the underwater coastal slope.

Initially, the content of PHEs obtained during the closest survey of long-term environmental monitoring (July 2014) was planned to be taken as the background content when analyzing the normalized values (to a fraction of less than 0.063 mm). The scale of this survey made it possible to estimate the contamination of clastic sediments, including those remoted from the influence of the coastal zone. But during the background survey, the Ni and Zn contents were not determined. Nevertheless, the median values of repeating elements (Cu, Cd, Pb, Hg, and OP) obtained during the background survey (July 2014) and survey of the coastal zone (May 2014) were quite close to each other. The median as well as the average, expresses the central or typical value in a set of data, but is weakly dependent on minimal and maximal values and outliers. Thus, the median of Cu content distribution by the results of the background environmental survey in July 2014 (12 values), normalized to the silty clay fraction, was 46.6 mg/kg, Hg –0.008 mg/kg, OP –29 mg/kg, Cd –1.33 mg/kg and Pb –3.01 mg/kg. Due to the proximity of the measured values for the further calculation of pollution indices the median values received in the coastal water area were taken as the background values. This approach does not contradict the methodology used in the work (Tomlinson et al., 1980).

### Statistics

Statistical methods have been applied to explain the relationships between the elements and a determination of their genesis. Thus, the close relationship of any HM with an obviously anthropogenic OP indicates that it was resulted from an economic activity too. Microsoft Excel 2007 and Statistical Package for the Social Sciences 10 (SPSS) software was used to perform statistical analyses. For the entire data set, the descriptive statistics and correlation coefficients between the elements were calculated. Cluster analysis was also used for investigating the similarities between HM in the sediments. A hierarchical cluster analysis was performed based on the complete linkage amalgamation rule.

### Assessment of the ecological state of the bottom sediments

Ecological indices for normalized pollution values were calculated to estimate the ecological state of bottom sediments. Contamination factor (CF) was estimated using the formula CF = C_Me_/C_Background_, where C_Me_ is the sampled element content, and C_background_ is the background element concentration (Hakanson, 1980). Modified degree of contamination (mCd) was calculated using the formula mCd = (CF1 + CF2 ... + CF_n_)/n, where n is the number of analysed elements (Abrahim, 2005; Abrahim & Parker, 2008). The Pollution Load Index (PLI) was determined according to (Tomlinson et al., 1980) with the formula PLI = (CF1 + CF2 ... + CF_n_)^1∕n^, where n is the number of analysed elements. The environmental risk assessment for each point was carried out using the potential environmental risk index (RI) according to the formula RI = E_r1_ + E_r2_ ... E_rn_; E_ri_ = CF_i_ * T_ri_ (Hakanson, 1980), where Er determines the potential ecological risk index for each HM, CF is the contamination factor of this element, and T_ri_ is Hakanson’s toxicity factors, which are 2, 5, 5, 30 and 40 for Zn, Pb, Cu, Cd and Hg, respectively. The CF index was calculated for HM and OP, the PLI, mCd and RI for HM only.

The index interpretation was done according to the classifications for CF and RI (Hakanson, 1980), mCd (Abrahim, 2005; Abrahim & Parker, 2008), PLI (Chakravarty & Patgiri, 2009).

### Sediment transport

To confirm the last (after last storm) significant direction of sediment transport on the linearly stretched part of the underwater coastal slope of the Curonian Spit (points 19–40), a method based on the determination of the changes in the grain size coefficients was applied (MacLaren & Bowles, 1985).

## Results

### Bottom sediments

Bottom sediments in the study area were mainly represented by sands from fine-grained to coarse-grained with an average median diameter of the sand fraction of 0.26 ± 0.36 mm. Coarse deposits (boulders, pebbles, gravels—sampling points 15, 16 and 18, respectively) were found in the root of the Curonian Spit. Such distribution is typical for the coastal zone with increased dynamics of sedimentary matter in the wave-surf zone (Atlas, 2010).

The total content of HM and OP in the bottom sediment was generally higher than the long-term average background values. Particular attention should be paid to the distribution of 5th (the highest) class of contamination (environmental quality standards for sediments) values of Cu (WGMS, 2003) in the Rybachy village area (points 28, 32, 34, 35, 38, and 40 with a content of 240, 170, 340, 150, 160, 240 mg/kg, respectively). Nearby the Curonian Spit during the environmental monitoring of the Kravtsovskoye oil field (D-6) Cu concentration values also belonged to the 4th and 5th classes (110 and 200 mg/kg in 2013, and were 110 and 300 mg/kg in 2014 at the points 7L and 6L, respectively). The Ni and Pb contents corresponded to the 5th class of contamination only at the points 32 (Ni –120 mg/kg) and 33 (Pb –130 mg/kg), Zn –4th class only at the point 33 (230 mg/kg). The highest concentrations of OP were recorded at the points 33 (650 mg/kg) and 35 (1,380 mg/kg), with an average value of 59.8 ± 45.9 mg/kg, which is far higher than background value (<40 mg/kg).

The main contradiction of the results is reduced to the spatial distribution of PHEs. The maximum PHEs content was found on the underwater coastal slope of the Curonian Spit, where there are no known potential sources of pollution (rivers, wastewater discharges, and industrial enterprises). Whereas the underwater coastal slope of the Sambia Peninsula—the most populated and economically developed coast (there is a port, the wastewater discharge system, and unorganized wastewater discharge), was cleaner (Table 1).

**Table 1:** The ratio of PHEs content in the bottom sediments of the underwater coastal slope of the Sambia Peninsula and the Curonian Spit.

Sambia Peninsula (points 1–17)				Curonian Spit (points 18–40)			
	Min.	Max.	Average	*σ*	Min.	Max.	Average	*σ*
Pollutant	mg/kg				mg/kg			
Hg	<0.005	0.007	<0.005	–	<0.005	0.01	0.005	0.002
Cd	<0.05	0.96	0.46	0.26	<0.05	0.86	0.40	0.23
Cu	0.98	56	25.3	18.6	20	340	98.7	84.7
Ni	0.67	12	5.41	4.02	<0.5	120	8.41	25.24
Pb	<0.5	19	1.71	4.64	<0.5	130	8.03	27.34
Zn	5.7	24	14.7	4.6	4	230	26.1	48.7
OP	<40	120	52.6	36.3	<40	1,380	151.8	305.4

### Coastal research

The average PHEs content on the beaches generally was noticeably lower than on the underwater coastal slope. The exception was Zn; its concentration was comparable to the bottom sediments. Predictably the beaches of the Sambia Peninsula were more polluted than the Curonian Spit ones (Table 2), due to anthropogenic load and the water runoff from settlements.

**Table 2:** The ratio of PHEs content on the beaches of Sambia Peninsula and the Curonian Spit.

Sambia Peninsula				Curonian Spit			
	Min.	Max.	Average	*σ*	Min.	Max.	Average	*σ*
Pollutant	mg/kg				mg/kg			
Hg	<0.005	<0.005	<0.005	–	<0.005	0.008	<0.005	–
Cd	0.08	0.54	0.24	0.14	0.061	0.39	0.16	0.08
Cu	0.88	89	21.2	24.4	<0.5	45	9.0	11.9
Ni	0.86	4.2	1.83	0.72	<0.5	4.8	1.7	1.6
Pb	<0.5	3.5	0.95	0.84	<0.5	5.8	0.96	1.24
Zn	2.1	190	19.8	44.2	1.4	8.1	4.4	2.0
OP	<40	72	<40	–	<40	50	<40	–

The average content of PHEs in the settlements of the Sambia Peninsula was significantly higher than on the beaches: Cd –0.53 ± 0.19 mg/kg; Cu –28.43 ± 7.76 mg/kg; Ni –2.63 ± 2.03 mg/kg; Pb –5.36 ± 4.44 mg/kg; Zn –28.43 ± 28.86; Hg –0.28 ± 0.51 mg/kg; OP –181.29 ± 157.76 mg/kg.

**Figure 2:**
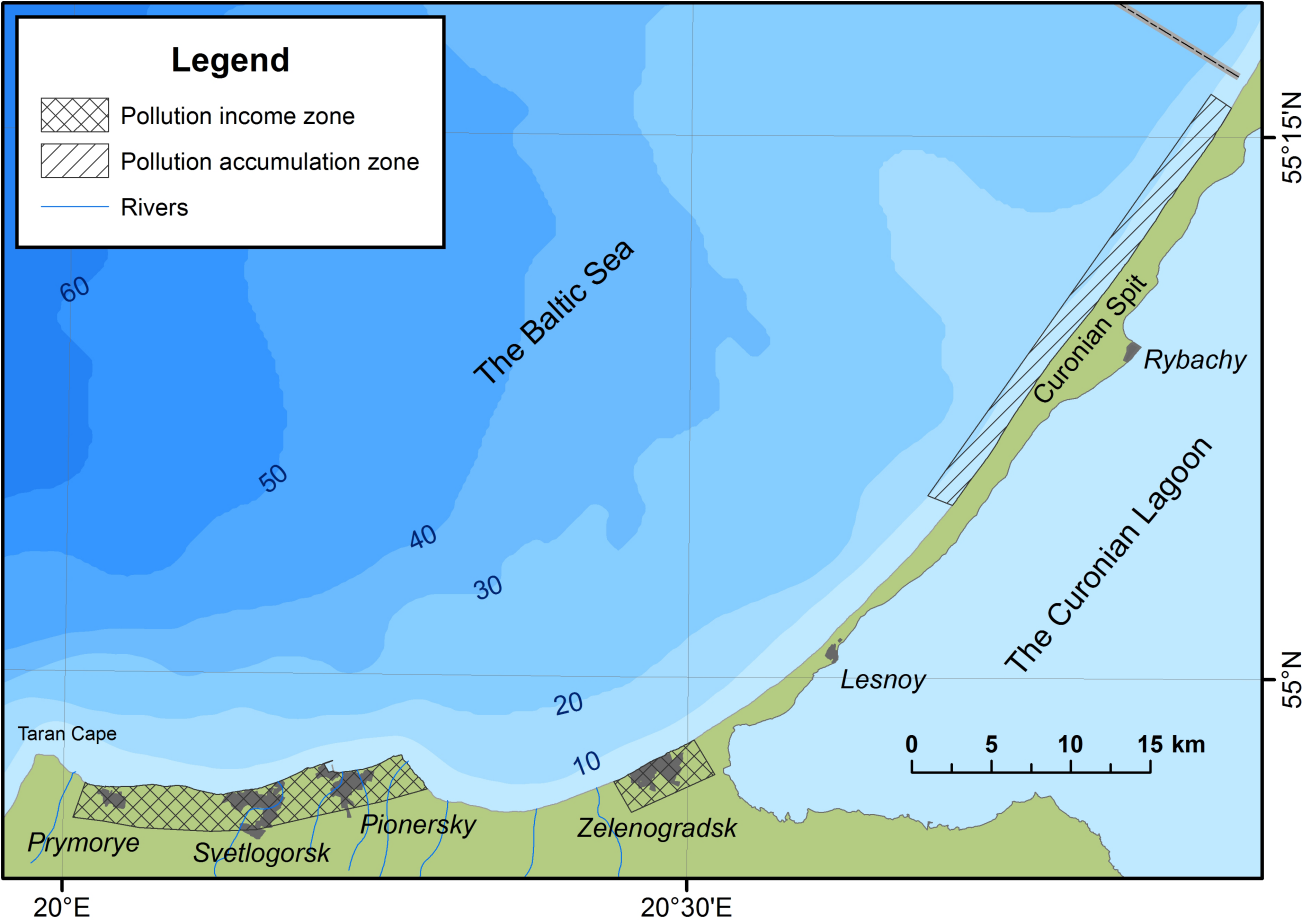
Areas of potential PHEs sources and accumulation.

Areas of potential PHEs sources (settlements on the northern coast of the Sambia Peninsula) and the area of pollution accumulation (the underwater coastal slope in the area of the Rybachy village) were identified by many PHEs (Fig. 2).

## Discussions

### Comparison with neighboring water areas

The content of Cd, Zn, Ni, Pb in the sediments of the studied area was comparable to the Polish coastal part and higher than in the territorial waters of Lithuania (Table 3). The average content of Cu was significantly higher than in the sediments of the Southern Baltic Sea. It should be kept in mind that the river runoff of the Vistula and the Oder rivers has a significant influence on the coastal zone of the Southern Baltic Sea. These rivers carry a large amount of HM from a heavily contaminated industrial areas of Poland, the Czech Republic, and Germany (Szefer et al., 2009). In this regard, the coastal zone of Lithuania seems to be more indicative.

**Table 3:** Average content of HM in the bottom sediments of the underwater coastal slope and their background values.

The studied area			
	Min.	Max.	Mean	*σ*	Polish coastal part	The territorial waters of Lithuania
Element	mg/kg				mg/kg	
Cd	<0.05	0.96	0.42	0.25	3.3^*^	0.04^***^
Cu	0.98	340	67.8	74.6	19.5^**^	1.4^***^
Ni	<0.5	120	7.1	19.2	27^**^	3.0
Pb	<0.5	130	5.4	21.0	37.5^**^	3.6^***^
Zn	4	230	13.7	13.5	87^**^	11.9^***^
Hg	<0.005	0.01	0.005	0.002	<0.02^****^	

**Notes.**

* Szefer et al., 2009 (average by the points 10, 13, 14, 15. 25, 26, 28).

** Belzunce Segarra et al, 2007 (at the point K-6)

*** Remeikaite-Nikiene et al., 2018 (the average for a shallow-water area from measurements at seven points).

**** Uścinowicz, 2011 (average for sands).

### Mineralogical composition of coastal sediments

The geological structure of the area both with modern active hydrodynamic factors determines the types and mineralogical composition of the sediments of the coastal zone (Emelyanov, 1986; Atlas, 2010; Krek, Stont & Ulyanova, 2016). The mineralogical composition of the sands is represented by minerals originating from the crystalline rocks of the Scandinavian Peninsula and local pre-Quaternary sedimentary rocks that have been processed as a result of erosion of the shore and the underwater coastal slope. According to X-ray structural phase analysis, the main minerals of sands are quartz and glauconite. Troilite FeS, chromite Fe (AlCr)_2_O_4_, ilmenite FeO.TiO_2_ or Fe TiO_3_, diopside CaMg (Si_2_O_6_), zircon have been recorded as minerals-admixtures (Information bulletin, 2014). The outcrops of the Neogene-Paleogene sands are traced in the coastal cliff and on the underwater coastal slope of the Sambia Peninsula (Dodonov, Namestnikov & Yakushova, 1976; Emelyanov, 1986). Glacial and modern marine deposits east of Gvardeisky Cape undergo the wave processing. Bed load especially under storm conditions can significantly change the contribution of sources to the overall mineralogical composition of bottom sediments (Krek, Stont & Ulyanova, 2016).

If the results of chemical analyzes is influenced by the mineralogical composition of clastic deposits (>0.063 mm), it would be expected to observe a direct dependence in the distribution of HM in areas with a predominance of different mineral sources for both beach and bottom sediments, taking into account their genetic similarity. Thus, on the northern coast there is a source of glauconite (Neogene-Paleogene deposits of the Sambia Peninsula) which is a good sorbent of natural and anthropogenically derived HM. Taking into account the sorption ability of glauconite and the absence of additional sources of contamination on the pathways of its transfer, it would be expected to observe higher PHEs contents in the area of its maximum concentration. However, the results of the study did not reveal such a relationship.

### Distribution of pollution along the coast

In coastal sediments a clear pattern was established—a reduce of PHEs concentration at a distance from sources of its entry (settlements, equipped places of vehicle access to the beach on the Sambia Peninsula). The beaches of the Curonian Spit remained clean (see Table 4), and the content of PHEs here was several times lower than in the bottom sediments. The peculiarity of the beaches is a good sorting of sand and the absence of a silty clay fraction (<0.063 mm only traces).

**Table 4:** The ratio of PHEs content on the beaches of the Sambia Peninsula and the Curonian Spit in terms of silty clay fraction.

Sambia Peninsula				Curonian Spit			
Min	Max	Average	*σ*	Min	Max	Average	*σ*	Median total for the coast
Pollutant	10^2^mg/kg				10^2^mg/kg			
Hg	0.001	0.083	0.009	0.02	0.001	0.038	0.008	0.009	0.002
Cd	0.01	10.19	**1.38**	2.41	0.3	1.3	0.46	0.40	0.41
Cu	1.70	868.9	95.59	205.5	9.9	401.7	**113.5**	105.2	50.6
Ni	0.56	272.67	**26.47**	64.44	0.007	123.7	9.15	26.02	3.01
Pb	0.10	50.06	**7.04**	16.09	0.1	213.72	11.16	45.28	0.53
Zn	2.16	625.79	**95.89**	177.92	0.01	378.12	34.68	78.42	13.9
OP	5.10	2,996.4	**261.2**	718.9	5.1	1,085.3	205.7	337.7	33.5

In the bottom sediments, the mineralogical composition of bedrock of the Sambia Peninsula did not have a significant effect on the redistribution of PHEs), so the authors took the pollution for anthropogenic. There are no significant concentrations of Cu, Pb, Zn, and Cd in the main natural minerals, although some of the minerals can be good PHEs sorbents. Another argument for the anthropogenic genesis of PHEs is the increased content of OP in the bottom sediments. Sources of seepage of oil hydrocarbons in the study area are unknown.

It is most likely that the significant influence on the distribution of PHEs has the presence of the fraction <0.063 mm with the highest sorption capacity in the grain size composition. (Szefer et al., 1995; Emelyanov, 1998; Pempkowiak et al., 1998; Pempkowiak, Sikora & Biernacka, 1999; Trofimov & Ziling, 2002; Beldowski & Pempkowiak, 2003; Zaborska, Winogradow & Pempkowiak, 2014).

Statistical analysis of the contents of HM and OP reduced to a silty clay fraction showed the comparability of the average values between the Sambia Peninsula and the Curonian Spit, what was not observed in the chemical analysis of samples. Offshore, the Curonian Spit only the average values of Cu and Pb exceeded the average values for the underwater coastal slope of the Sambia Peninsula (Table 4).

The silty clay fraction of the sediments of the coastal zones is more polluted than the clay fraction of the Gdansk Deep. At a background point 22L (which is remotely situated from the coast), where the percentage of the silty clay fraction was more than 95%, in July 2014 the Hg content was 0.1 mg/kg, Cd –1.9 mg/kg, Cu –77 mg/kg, Pb –63 mg/kg, OP –188 mg/kg. The results obtained at this point are fairly close to the results obtained for the Polish part of the Gdansk Deep (point P1) (see Fig. 1). The Pb content was estimated at 82 mg/kg (Zalewska et al., 2015) and 75 mg/kg (Szefer et al., 2009), Cd and Hg 0.17 and 0.05 mg/kg, respectively (Zalewska et al., 2015). Similar results for Cd for surface sediments were obtained −1.51 mg/kg (Pempkowiak, 1991) and −1.7 mg/kg (Glasby et al., 2004).

**Figure 3:**
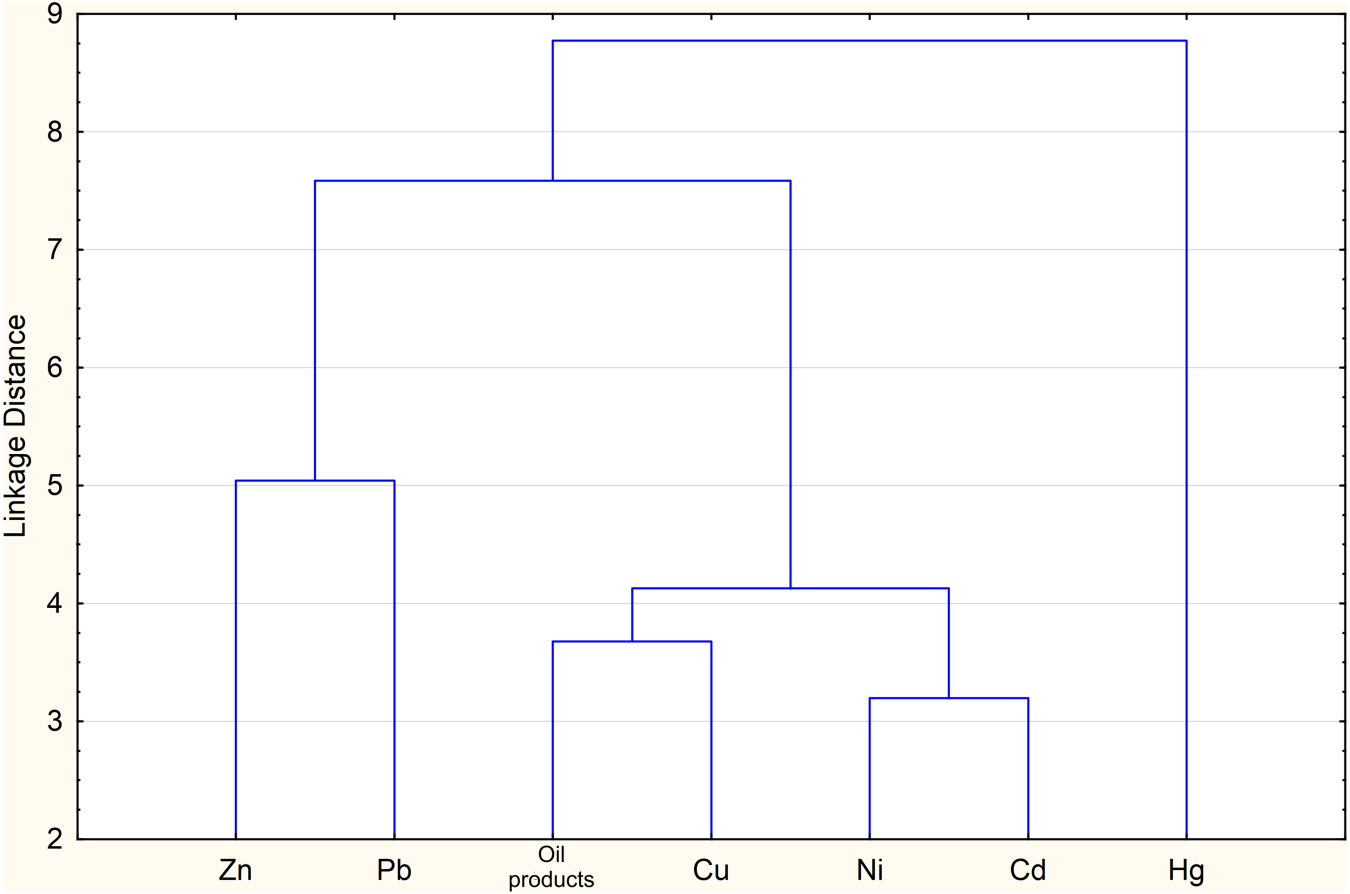
Dendrogram obtained by cluster analysis of the sediment samples.

High correlation in the silty clay fraction revealed by cluster analysis was noticed for Ni-Cd, Cd-Cu and Cu-Ni (Table 5). The high correlation of OP-Cu, OP-Cd, and OP-Ni indicate a similar pathway of income of microelement with certainly anthropogenic OP (Fig. 3). The distribution of Hg does not correlate with other pollutants, which is probably due to its genesis.

**Table 5:** Correlation matrix between the elements, reduced to the silty clay fraction. High correlation is shown in bold.

	Hg	Cd	Cu	Ni	Pb	Zn	OP
Hg	1.00	0.12	0.04	−0.07	−0.03	0.00	0.03
Cd	0.12	1.00	**0.76**	**0.86**	0.20	0.50	**0.84**
Cu	0.04	**0.76**	1.00	**0.79**	0.20	0.55	**0.81**
Ni	−0.07	**0.86**	0.79	1.00	0.23	0.55	**0.80**
Pb	−0.03	0.20	0.20	0.23	1.00	0.65	0.42
Zn	0.00	0.50	0.55	0.55	0.65	1.00	0.56
OP	0.03	**0.84**	**0.81**	**0.80**	0.42	0.56	1.00

### Ecological indices

**Figure 4:**
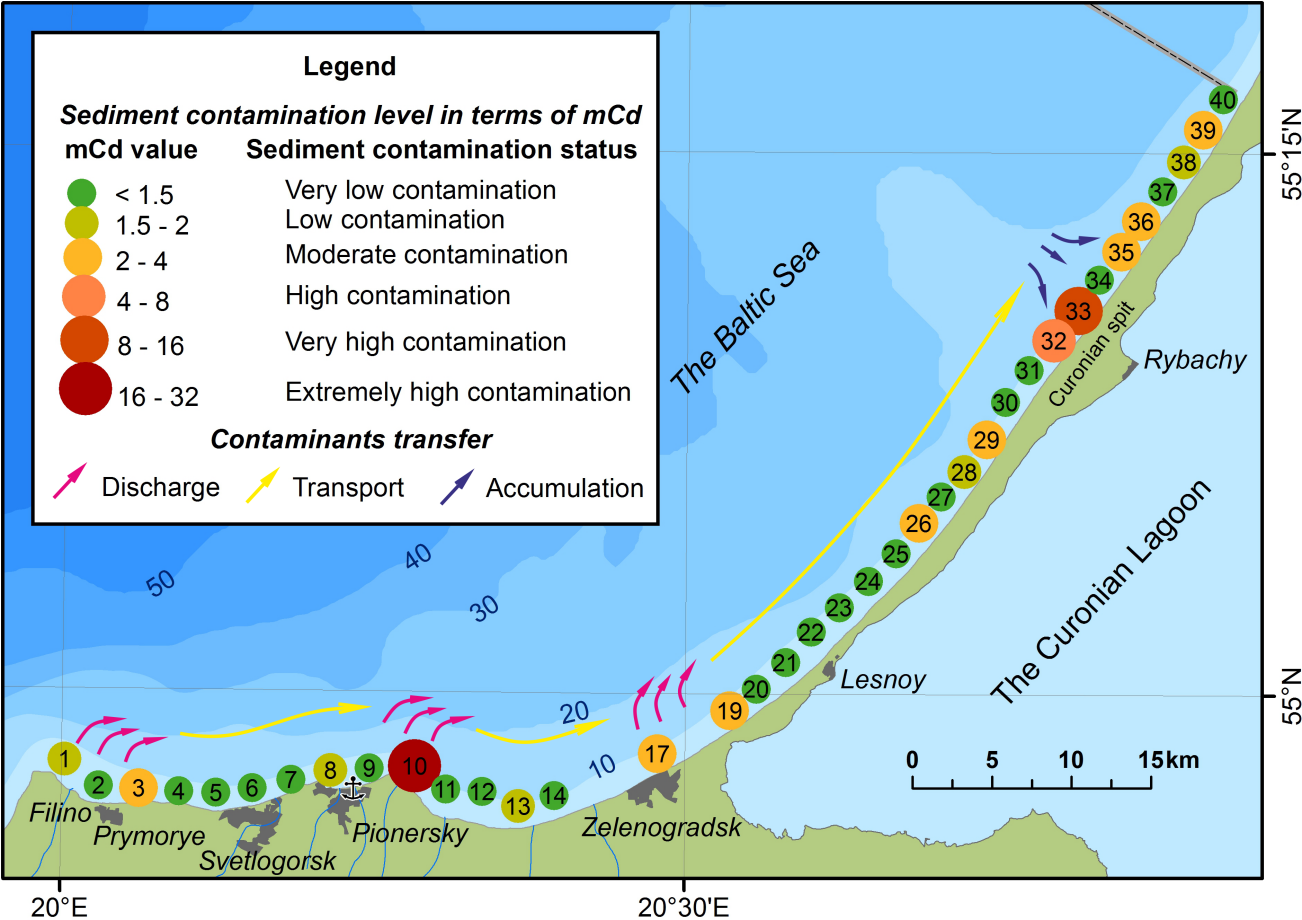
Spatial distribution of the integral index mCd.

The CF coefficient showed a high degree of pollution by separate indicators, mainly in three areas: the points 1 and 3 correspond to the area of the vehicle access to the beach and the unorganized drainage of Filino and Primorye settlements; the point 10 corresponds to the organized place of discharge of water sewage treatment systems; the point 17 is located close to the city of Zelenogradsk. Nevertheless, even after the reduction to the silty clay fraction, an anomalous area offshore the Rybachy (point 33) took place (Table 6). In principle, all areas are allocated indices, applied only to HM, the most indicative of which is mCd (Fig. 4).

**Table 6:** Indexes CF, mCd, PLI, and RI.

CF						
Point	Hg	Cd	Cu	Ni	Pb	Zn	OP	mCd	PLI	RI
1	**36.58**	**6.05**	0.25	3.07	0.19	5.33	0.15	**8.6**	**2.4**	**1,652**
2	0.61	0.40	0.17	0.18	0.19	0.27	0.15	0.3	0.3	38
3	0.27	0.04	3.54	**9.28**	**94.38**	**44.91**	0.15	**25.4**	**3.3**	***546***
4	0.27	**6.15**	1.22	2.64	0.19	4.12	0.15	2.4	**1.3**	207
5	1.42	1.17	0.47	0.65	0.19	0.70	0.15	0.8	0.6	96
6	1.91	1.92	0.91	0.71	1.00	1.00	0.15	1.2	**1.2**	144
7	0.27	1.06	0.06	0.82	0.19	0.69	0.15	0.5	0.3	44
8	0.27	1.53	0.91	5.42	1.80	3.16	**6.58**	2.2	**1.5**	73
9	0.27	0.45	0.14	1.70	**21.12**	0.42	2.11	***4.0***	0.8	131
10	0.27	**24.91**	**17.17**	**90.51**	**90.38**	**34.41**	**89.48**	**42.9**	**17.9**	**1,330**
11	0.27	0.30	0.31	0.95	1.06	0.30	0.44	0.5	0.4	27
12	0.27	0.39	0.29	1.49	0.19	0.58	0.80	0.5	0.4	25
13	0.27	3.24	1.86	**8.13**	0.19	2.64	3.23	2.7	**1.4**	121
14	0.27	0.14	0.03	0.48	0.19	0.15	0.31	0.2	0.2	16
17	**19.08**	3.09	1.00	5.78	0.19	4.54	**12.99**	***5.6***	**2.6**	**866**
19	**7.25**	2.96	1.90	1.55	3.11	1.82	**9.05**	3.1	**2.7**	***406***
20	0.27	1.03	0.37	0.31	0.63	0.50	0.15	0.5	0.5	47
21	0.27	0.67	0.20	0.26	0.86	0.39	0.83	0.4	0.4	36
22	0.27	0.19	0.21	0.04	0.38	1.09	0.15	0.4	0.2	20
23	0.54	0.16	0.30	0.02	1.00	0.12	0.15	0.4	0.2	33
24	1.00	0.63	0.40	0.19	0.99	0.38	0.15	0.6	0.5	66
25	2.09	0.64	0.44	0.19	1.46	0.26	0.15	0.8	0.6	112
26	**6.50**	3.19	1.52	1.08	**6.71**	1.87	4.25	3.5	**2.7**	***399***
27	**6.32**	0.11	2.78	0.78	0.19	1.68	0.15	2.0	0.9	273
28	3.96	0.98	**7.94**	1.00	0.19	2.88	2.05	2.8	**1.6**	232
29	**16.71**	0.26	4.93	1.55	0.19	3.65	**6.07**	***4.5***	**1.7**	**705**
30	0.27	0.36	0.88	0.12	2.82	0.28	0.60	0.8	0.4	40
31	1.90	0.07	0.94	2.39	2.17	0.88	1.94	1.4	0.9	94
32	2.40	1.41	3.46	**41.06**	**11.47**	1.41	4.31	**10.2**	**4.4**	214
33	4.40	2.69	2.34	**7.09**	**402.90**	**27.14**	**31.91**	**74.4**	**11.4**	**2,310**
34	0.62	0.50	1.61	0.87	3.61	0.34	1.00	1.3	0.9	66
35	3.45	1.00	2.33	2.87	**7.86**	0.56	32.41	3.0	**2.2**	219
36	**12.10**	2.41	**6.93**	1.83	0.19	4.24	25.90	***4.6***	**2.6**	***596***
37	0.27	0.45	2.48	2.58	0.19	2.65	5.85	1.4	0.9	40
38	2.34	1.10	2.91	0.29	**6.07**	0.92	2.22	2.3	**1.5**	172
39	5.05	2.03	2.36	0.69	**8.44**	1.68	4.39	3.4	**2.5**	***319***
40	2.43	2.10	2.10	0.02	1.40	0.01	1.42	1.3	0.2	178

**Notes.**

A very high level of contamination is shown in bold, italics are high.

### Transport of pollution

The PHEs content depends on lithodynamic conditions of the underwater coastal slope. The bottom erosion is most active on the Sambia Peninsula. The bottom erosion is most active on the coastal slope of the Sambia Peninsula. Accumulation processes prevail offshore the middle part of the Curonian Spit what contribute to the accumulation of PHEs. The nature of hightened concentrations of the PHEs here is related to alongshore transport of the sediments (Krek, Stont & Ulyanova, 2016), which depends on the hydrodynamic situation. The existence of an alongshore bed load transport directed from Taran Cape along the Curonian Spit was confirmed by many authors (Shuisky, 1969; Shuisky, Boldyrev & Kochetkov, 1970; Ryabkova, 1987; Bogdanov et al, 1989; Leontyev, Zhindarev & Ryabkova, 1989). The zones of convergence of various spatial and temporal scales were observed in the middle part of the Curonian Spit and corresponded to the accumulation zones.

The results of wave modeling (Soomere & Viška, 2013) confirm the weakening of the alongshore bed load and the appearance of a convergence zone in the designated area. Nevertheless, the general direction of the material transport can vary depending on certain hydrometeorological conditions.

Based on the results of the counting of the grain size characteristics reflecting the last direction of material transport, the most probable direction of the sediment transport from the root of the Curonian Spit along its coast in the northeasterly direction was established (*Z* = 7.98, significance level is 0.01, by MacLaren & Bowles, 1985). The direction of such transport allows us to assume input of pollution from the Sambia Peninsula.

The distribution of pollution is divided into 3 main zones: 1 - Contamination zone, 2 - Transit zone, and 3 - Pollution accumulation zone. The locations of the transit and the accumulation zones are not constant and can vary depending on the intensity of the alongshore bed load (see Fig. 4).

## Conclusion

The method used of pollution reduction to the silty clay fraction allowed to allocate the sources of anthropogenic impact to the bottom sediments, which could not be performed directly from natural data. The general distribution of HM and OP indicates a high anthropogenic pressure on the coastal zone, which is not consolidated only among pollution sources, and can spread to sufficiently remote and unique areas. The silty clay fraction of the coastal sediments is more polluted than the silty clay fraction of the Gdansk Deep. Nevertheless, the beaches seem to play a transitory role in the distribution of pollution, since fine-dispersed particles are carried out to the water area when sand is washed up as a result of wave-surf activity. The pollutants were moved from their sources to the accumulation zone on the underwater coastal slope of the Curonian Spit by alongshore sediment transport. Apparently, alongshore transport is the determining factor in the redistribution of anthropogenic load in the coastal zone of the Kaliningrad Region.

##  Supplemental Information

10.7717/peerj.4770/supp-1 Supplemental Information 1 Background values

10.7717/peerj.4770/supp-2 Supplemental Information 2 MD and SO

10.7717/peerj.4770/supp-3 Supplemental Information 3 Pollution beach sediments

10.7717/peerj.4770/supp-4 Supplemental Information 4 Grain size

10.7717/peerj.4770/supp-5 Supplemental Information 5 Pollution bottom sediments

## Additional Information and Declarations

### Data Availability

The following information was supplied regarding data availability: The raw data are provided in the Supplemental Files.
